# Supplementary material for: Magnitude and determinants of appropriate complementary feeding practice among mothers of children age 6–23 months in Western Ethiopia
Source: PLoS One. 2020 Dec 31;15(12):e0244277. doi: 10.1371/journal.pone.0244277 (PMC7774947; doi:10.1371/journal.pone.0244277)
Supplement: S1 File — (DOC) [file pone.0244277.s001.doc]

| BAHIR DAR UNIVERSITY  Faculty of Chemical and Food engineering  Department of Applied Human nutrition  Questionnaire (English)  ID No of the Questionnaire: ________ | | | |
| --- | --- | --- | --- |
| Questionnaire for mothers/primary caregivers of children 0-23 months old | | | |
| **Section 01: Survey General Information** | | | |
| N^o^ | Questions and Explanations | Alternatives/Answers | Skip guide |
| 01 | Questionnaire number consist of the Region and Woreda code, Kebele & Household Number  (To be filled before interview) | Questionnaire N^o^ |_____|_____|_____|_____|  Region _______Kebele name_______  Woreda_____House number _______ |  |
| 02 | Interviewer and field supervisor name & signature  Interviewer: | Name፡ _________Signature፡ ________  Name፡ __________Signature፡ ________ |  |
|  |  |  |  |
|  | Field supervisor : |  |  |
| 03 | Number of days the household was visited for interview | One (01)  Two (02)  Three (03) |  |
| 04 | Date the questionnaire started and completed |  |  |
|  | Date interview started: | [_____|_____|_____|  dd MM YY |  |
|  | Date interview completed: | [_____|_____|_____|  DD MM AA |  |
| 05 | Questionnaire status | Completed (01)  Absent at Home (02)  Unwilling to Participate (03)  Unidentified House (04)  Other (Specify)___________________ (05) |  |
| **Introduction and consent form** | | | |
| Information and consent  Dear respondent,  Good morning/Good afternoon. Thank you for being willing to talk to me. This study is intended to determine “Magnitude and associated factors of appropriate complementary feeding practice among mothers of children aged 6-23 months in Horo Guduru Wellega, East Wellega, Oromia regional state”. The purpose of my visit is to take information from you on the aforementioned issue. If you are willing to participate in the study, I will ask you few questions for 20-30 minutes. Your name will not be written on this form and will never be used in connection with any of your information. You do not have to answer any question that you are not comfortable with, and you may end the interview any time you want to. However, your honest answers to these questions will help us for better understanding of child feeding practice in your locality, and will eventually help in designing and implementing appropriate interventions to alleviate the problem. Hence, we greatly appreciate your participation in this study.  Problems connected with the study፡  There is no risk to your child and he/she would not experience any discomfort at all.  Involvement compensation፡  By involving in the study, you wouldn’t get any payment.  Confidentiality  No matter what information you provide, it will be kept strictly confidential and your views will not be shown to other family members or relatives. In the questionnaire, we will use your code rather than your name. There will not be any identifying information about you or your name in the survey responses. Similarly, when the survey finding is communicated or published, information that may identify you or your name will not be used. Any information that may identify you will be kept out of the questionnaire and your views will be kept in secret to be used only by the researchers to organize the study result.  Interruption of voluntary participation  Participation in this survey is completely voluntary and you can choose not to talk with me, not to answer any/all the questions or command me to leave your home. You may stop participation at any time. There is no right or wrong answer for the questions, what we need to know is your view and experience. You can even make your views not to be included in the study after the interview is completed.   - If you have any questions about the survey, I am ready to respond. - Do you have any questions about the survey? Please let me know if anything I have stated is not clear and I will be happy to explain it further to ensure you understand.   _____________________________ ___________ __________  Witness Signature (if only the participant is unable to write) Date Time   | Are you willing to participate in the study? | | --- |   Yes_______ No__________  After the participant’s verbal consent is taken , confirm it by check box  _____________________________ ___________ __________  Signature of a person who take the consent Date Time  _______________________________  Name of the person who take the consent  ____________________  Interviewee code: | | | |

| **Section 02፡ Background information about the mother/primary caregiver and socio-economic information about the household** | | | | | |
| --- | --- | --- | --- | --- | --- |
| N^O^ | **Questions and Explanations** | **Alternatives/Answers** | **Skip Guide** | | |
| 06 | Who is responsible for child/ Who takes care of the child? | Mother(01)  Grand-mother(02)  Sister(03)  Servant(04) |  | | |
| 07 | What is your religion? | Orthodox Christian (01)  Protestant (02)  Wakefata (03)  Muslim (04)  Non-believer (05)  Other (specify) __________________ (06) |  | | |
| 08 | What is your ethnicity? | Oromo(01)  Amhara(02)  Gurage(03)  Others (specify)__________(04) |  | | |
| 09 | Residence? | Urban(01)  Rural(02) |  | | |
| 10 | How old are you?  (Age in completed years) | ……………….. |  | | |
| 11 | What is your current marital status? | Married/Living together(01)  Single (02)  Widowed (03)  Divorced (04)  Separated (05) |  | | |
| 12 | How many family members are there in your home? | …………………….. |  | | |
| 13 | Birth order | ……………………. | If a 1^st^ child skip to Q16 | | |
| 14 | If the child is not the first for you; in what birth interval (year) do you give birth? | …………………………… |  | | |
| 15 | What is the sex of the child? | Male(01)  Female(02) |  | | |
| 16 | What is the age of the child? | ...................... |  | | |
| 17 | What is your highest educational status? | No formal education (01)  Grade 1 – 8(02)  Grade 9 – 12(03)  Diploma and above(04) |  | | |
| 18 | What is your occupation? | Farmer/Agricultural worker (01)  Daily laborer (02)  Trader (03)  Employee (04)  House wife (05)  Other (specify) _____________ (06) |  | | |
| 19 | What is the occupation of your  husband? | Farmer/ Agricultural worker (01)  Employee(02)  Daily laborer (03)  Trader (04)  Other (specify) _______________ (05) |  | | |
| 20 | Husband educational status | No formal education (01)  Grade 1 – 8(02)  Grade 9 – 12(03)  Diploma and above(04) |  | | |
| 21 | Monthly Income of the household? **(in ETB)?** | 0-999(01)  1000-1999(02)  2000-2999(03)  >3000(04) |  | | |
| **Section 3: Health service related questions** | | | | | |
| 22 | Did you visit health facility for ANC during your pregnancy for this child? | Yes(01) | | >24 | |
|  |  | No(02) | |  | |
| 23 | If your answer is **yes** to Q-22, how many times did you receive ANC (number of antenatal care)? | ……………………… | |  | |
| 24 | Where did you give birth to  this child /place of delivery/ | Home(01)  Hospital(02)  Health Center(03)  Other (specify)___(04) | |  | |
| 25 | Have you received post natal care within the first 7 days? | Yes(01)  No(02) | |  | |
| 26 | Did you receive advice when taking your child to health facility when he/she was sick? | Yes(01)  No(02) | |  | |
| **Section 04:Knowledge based questions** | | | | | |
| 27 | Did you get health education on complementary feeding at any time of your visit to a health facility? | Yes(01) | | |  |
|  |  | No(02) | | | >Q28 |
| 28 | If your answer is yes to Q-27 ,what was the information that you acquired during your visit(more than one answer is possible | To start complementary food alongside breast milk at 6 month(01)  The type and diversity of food that should be given to the child(02)  The consistency of the food(03)  The amount and frequency of feeding(04)  The safety and hygiene of complementary foods(05)  How to feed your child actively(06)  To continue breast feeding till two years(07)  To continue feeding during illness and feed more after illness(08)  others(specify)-----------------------(09) | | |  |
| 29 | Have you received advice on CF when you went to a health facility to get immunization service for your child? | Yes(01)  No(02) | | |  |
| 30 | If your answer for Q-29 is yes, what was the source of nutrition information about complementary feeding for your child? | Health professional(01) | | |  |
|  |  | Mass media(02) | | | >Q31 |
|  |  | Peer-group(03)  Health development army(04) | | |  |
| 31 | If your answer is mass media, which one? | Radio(01)  Television(02)  Newspaper(03)  Internet access(04) | | |  |
| **Section 05: Practice based questions** | | | | | |
| 32 | Have you started complementary foods for your child? | Yes (01) | | |  |
|  |  | No(02) | | | >Q36 |
| 33 | At what age did you start giving him/her these foods? | < 6 month(01)  at 6 month(02) | | | >Q34 |
|  |  | after 6 month(03) | | | >Q 35 |
| 34 | Why you start before (early) six months? | Due to my occupation outside, I haven’t time to give breast milk(01)  Due to insufficient of breast milk(02)  Due to community influence (03)  Due to existence of large family in house.(04)  Due to lack of knowledge/information on time of initiation (05)  Medical and breast illness(06) | | |  |
| 35 | If the response is after six month, ask why she started late? | Due to lack of money (01)  Due to lack of food in the house(02)  Due to lack of knowledge on when infant / child start complementary food (03)  Due to community influence(04) | | |  |
| 36 | What was the reason for not giving additional foods at six month?  (Ask, If the respondent response after six month) | Mother felt breast milk alone was sufficient(01)  Mother think the child is young to start  complementary foods(02)  Mother think that the child may be chocked (03)  Others(specify)(04) | | |  |
| 37 | If your answer is yes for Q **-**32, what was the additional food or fluid that you gave in the past 24 hour? | Cow’s milk(01)  Sugar solution(02)  Formula milk(03)  Porridge(04)  Injera with wot(05)  Fruit and vegetable porridge(06)  Other (specify)______________(07) | | |  |
| 38 | How many times in a day do you feed the child additional food or fluid other than breast milk? | __________ Times | | |  |
| 39 | How do you feed the complementary food to your child? | Based on schedule(01)  Based on demand(02) | | |  |
| 40 | If the response is based on demand, what is the possible reason not to feed the child based on schedule? | Cultural(01)  Lack of time (02)  Lack of knowledge(03)  Lack of caregiver(04)  Health problem (05) | | |  |
| 41 | Who is responsible to feed the child? | Caregiver(01)  Husband(02)  Grandmother(03)  Parent (04) | | |  |

| 42 | Which method do you use for feeding? | Spoon(01)  Hand(02)  Bottle(03)  Combination of above(04) |  |
| --- | --- | --- | --- |
| 43 | What are the main sources of complementary food for your child? | Cereals (01)  Pulse(02)  cereal and pulse(03)  cereal, pulse and nuts(04)  cereals, pulse, nuts and dairy product(04)  Root and tubers(05)  Egg (05)  Meat (06)  Others(07) |  |
| 44 | Do you feed your child complementary food prepared from animal product mixed with plant product during fasting? | Yes(01)  No(02) | >Q45 |
| 45 | If you do not prepare, what is the main reason for not prepare it? | Due to religion(01)  Due to cultural reason (02)  Due to contamination of material(03)  Due to lack of knowledge (04)  There is no budget, only for child (05) |  |
| 46 | Do you prepare complementary foods from both meat and plant products? | Yes(01) | >Q47 |
|  |  | No(02) |  |
| 47 | If your answer for question number 46 is yes, in which form (texture) do you prepare the food? | Thin like gruel(01)  Thick like porridge (02)  Solid foods(03) |  |
| 48 | Do you use factory processed foods as complementary food? | Yes(01) |  |
|  |  | No(02) |  |
| 49 | If your answer for Q-48 is yes, which type of processed food do you use? | Powdered milk(01)  Pasteurized milk (02)  Cereal based(03)  Fruit based (mango juice etc.) (04) |  |

# **ANNEX II**

**Dhaabbata Techonolojii Bahir Daritti**

**Kutaa barumsaa” Chemical and Food Engineering”**

Sadarkaa sirna nyaata madaalamaa Haadholiin Daa’imman Umrii isaanii ji’a Jahaa hanga waggaa lama jidduu tahan qabanii qopheessuuf shaakalanii fi dhimoota murtessa isaa ta’aan sadarkaa mana manatti funaanuuf, aanaa Horroo, Naannoo Oromiyaa, dhiha Itoophiyaa.

# **Afaan Oromo Version of Informed Consent Form**

**Waligaltee**

Mata duree: sadarkaa sirna Harma qofaa hoosisuu Haadholii Daa’imman Umrii ji’a Jahaa gadi tahan qabanii fi dhimoota murtessa issa ta’aan sadarkaa manaa manatti funaanuuf, aanaa Horroo, Naannoo Oromiyaa, dhiha Itoophiyaa.

Hogganaa Qorannichaa: Dabalaa Dhaabaa

Teessoo: Dhaabbata Techonolojii Bahir Daritti, Muummee barnootaa Applied Human Nutrition

**Odeeffannoo Wali gala Waa’ee Qorannichaa**

Ati fi da’imnikee qorannoo waa’ee harma qofaa hoosisuu fi dhimmoota muteessaa isaa adda baasuu jedhu irratti akka hirmaattaniif afferamtaniittu. Qorannoon kun baay’ee barbaachisaa fi haadhoolii harma hoosisan gorsuuf gargaara.

Qorannoo kana irratti hirmaachuuf fedha yoo qabaattaniif dhimmoota dhuunfaa keessanii kan akka Umurii,sadarkaa barnootaa, bakka jireenyaa, baay’ina miseensa maatii keessanii fi akkasumas dhimmootaa waa’ee Daa’ima keessanii gosa dhalootaa, Da’ima meeqaffa akka isniif ta’e, umurii; dabalataanis Gargaarsa fayyaa kan akka hordoffi yeroo ulfaa, Da’umsa boodaa fi gorsa waa’ee harma hoosisuu fudhachuu keessan isin gaafanna. Kanaafis yeroo keessan daqiiqaa 30- 45 isinitti fudhachuu danda’a.

**Rakkoolee isin Mudachuu Danda’an**

Isiniif fi mucaa keessan irratti mudhaan qaqabu hin jiru.Garuu gaaffi qorannoo kana deebisuuf yeroo keessan aarsaa gochuu isin gaafata.Akkasumas gaaffileen tokko tokko isinitti toluu dhiisuu danda’u. Isin garuu gaaffi isinitti hin tolle dhiisuu ykn gidduutti addaan kutuu fi kan feetan qofa deebisuuf mirgi keessan eegamaadha.

**Hogganaa nama daataa funaanuu**

- Gaaffiin isiniif hin galle yoo jiraate ani isiniif deebisuuf qophaa’aa dha.
- Gaaffii ifa isiniif hin taane qabduu? Na gaafadhaa

Mallattoo namni gaafatuu walii galtee isaa afaaniin mirkaneessu isaa.

Maqaa nama ragaa funaanee: ………………………………………

Mallattoo nama ragaa funaanee: ………………………………….

Guyyaa: …………………………………

Hirmaachuudhaaf fedhii qabduu? Eeyyee---------------- lakki/miti------------------------

___________________________________ _____________

Maqaa fi Mallattoo nama yeroo walii galteen kun raawwate argee Guyyaa

Lakk eenyummaa Qoranoof kenname: _______________

**Dhaabbata Techonolojii Bahir Daritti**

**Muummee Barnoota Applied Human Nutrition**

Sadarkaa sirna nyaata madaalamaa Haadholiin Daa’imma Umrii ji’a 6 hanga 23 tahanif qopheessanii fi dhimoota murtessa isaa ta’aan sadarkaa mana manatti funaanuuf, aanaa Horroo, Naannoo Oromiyaa, dhiha Itoophiyaa,

# Afaan Oromo version questionnaire

Gaaffiillee qorannoo Afaan Oromootiin qophaa’e

| **Section 01: Funaansa odeeffanoo walii gala** | | |
| --- | --- | --- |
| **Lakk** | **Gaaffii fi Ibsa** | **Filannoo/Deebii** |
| **01** | Maqaa Naannoo,Aanaa, gandaa fi lakkoofsa manaa  **(***osoo gara gaafii fi deebii waa’ee qoranootti hin galiin dura bakka duwwaan fulduraa kana guutaa)* | Naannoo: oromiyaa  Aanaa: Horroo  Ganda…….………………..  Lakk. Manaa: ……… |
| **02** | Maqaa Namaa odeeffannoo funanee/ nama gaafii fi deebii ummata waliin gaggeesse  Maqaa ogeesa oddeefanoo funanee:  Hoganaa odeefanoo funanee : | Maqaa-------------------------------  Mallattoo፡ ________  MAqaa ________________  Mallattoo፡ ________ |
| **03** | **Baay’ina guyyaa Manichi Gaaffiidhaaf Hordofame** | **Guyyaa Tokko (01)**  **“ Lama (02)**  **“ sadii ( 03)** |
| **04** | Guyyaa odeeffanoo funanuu eegalee fi guyyaa itti xumuree  Guyyaa odeeffanoo funanuu eegalee  Guyyaa itti xumuree : | [_____|_____|_____|  Guyyaa ji’aa bara  [_____|_____|_____|  Guyyaa ji’aa bara |
| **05** | Haala Gaafiillee qoranoo  : | Xumuraniru(01)  Hin xumuree (02)  Hirmachuuf fedhii hin qaban (03)  Sababa biroon yoo jiraate sanduuqa keessatti guuti ( ___________________) (04) |

| **KUTAA TOKKOFFAA: GAAFFILEE HAWASSUMAA FI DINAGDEE** | | | |
| --- | --- | --- | --- |
| Lakk | **Gaaffii fi Ibsa** | Deebii | Adeemsa |
| 06 | Itti gafatamummaan daa’ima kan kununsuu eenyuudha? | Haadha(01)  Akkoo/Haadha Abbaa(02)  Maatii(03)  Obbooleetii(04)  Hojeetuu(05)  Kan biroo ………………….(06) |  |
| 07 | Amantaan keessan maaliidha? | Ortodoksii (01)  Protestantii (02)  Waaqefataa (03)  Musliima(04)  Amantaa hin qabu (05)  Kan biro(ibsi) __________________ (06 |  |
| 08 | Qomoon keessan maaliidha? | Oromoo(01)  Amharaa(02)  Guragee(03)  Kan biiroo(ibsi)------------ ---------(04) |  |
| 09 | Bakki jireenyaa Keessanii eessa? | Baadiyaa(01)  Magaala(02) |  |
| 10 | Umriin keessan meeqadha?  *(Bakka duwwan guutaa)* | -------------------- |  |
| 11 | Haalli gaa’ela keessanii haala akkami irra jira? | Abbaa manaa waliin jirra(01)  Dhuunfaakooti/Hin heerumne (02)  Fooniin addunyaa kanarra boqotan(03)  Wal hiikne (04)  Bakka adda addaa jirra (05) |  |
| 12 | Sadarkaa barumsaa isiin irra geessan maaliidha? | Barreessuus Dubbisuus hin danda’u(01)  Barreessuf dubbisuu qofa(02)  Kutaa 1-8 (03)  Kutaa 9-12 (04)  Diploomaa fi isaa ol(05) |  |
| 13 | Miseensi maatii meeqa qabdu? |  |  |
| 14 | Daa’imni keessan kun isinif meeqaffaadha? | ---------------------- | Daa’imma jalqabaa miti yoo ta’e gaaffii itti aanutti ce’i |
| 15 | Da’imni kun isiniif isa jalqabaa yoo hin taane; daa’ima kana duraa deessanii turtii yeroo hammamii booda daa’ima kan deessan? | --------- |  |
| 16 | Saalli Daa’ima keessanii maali | Dhiira (01)  Dhalaa(02) |  |
| 17 | Umrii daa’ima kanaa meeqa? | ------------------ |  |
| 18 | Sadarkaan barnoota keetii akkami? | Barumsa idilee hin qabu/hin baranne(01)  Kutaa 1-8 (02)  Kutaa 9-12 (03)  Diploomaa fi isaa ol(04) |  |
| 19 | Gosti hojii kee maali? | Qonnaarratti kan bobateedha(01)  Humnan bulaa (02)  Daldaltuu(03)  Hojjettuu mootummaa(04)  Hojii manaa(05) |  |
| 20 | Maddi galii keessanii maali dha? | Loon, Re’ee, Hoolaa fi k.k.f. gurguruudhaani(01)  Lafa gurguruu/ kirayeessuudhaani(02)  Meeshaalee garaagaraa kan mana keessaa gurguruutiin(03)  Midhaan gurguruudhaan(04)  Hojii humnaa hojjechuudhaan(05)  Suuqii/daldala(06)  Miindaa ji’aa(07)  Kan biro------------------------------(08) |  |
| 21 | Gaaffii 18^ffaa^  irratti hundaa’uun hammi galii keessanii ji’aan meeqa ta’a? | ---------------- |  |
| **Kutaa lammaffaa:Gaaffiilee Tajaajila fayyaa Haadhaa fi Mucaa waliin wal qabate.** | | | |
| 22 | Yeroo garaatti battu wajjira fayyaa adeemuun gorsa ogeesaa fudhachaa turtanii? | Eeyyee(01)  Miti (02 | Miti yoo jette gara G-25ti darbi |
| 23 | Yoo eeyyeen jettan, yeroo meeqaaf mana yaalatti deddebi’uun gorsa fudhachaa turta? | --------- |  |
| 24 | Yeroo hordoffii Da’umsaa taasisaa turtetti Barnoonni/gorsi waa’ee harma hoosisuu siif kennameeraa? | Eyyee(01)  Miti/Lakki (02) |  |
| 25 | Daa’ima ammaa kana eesatti deessan? | Mana jireenya (01)  Mana yaalaa (02)  Kan biraa (Ibsi)____________ (03) |  |
| 26 | Haalli da’umsa keessanii akkam ture? | Nageenyumaan deesse(01)  Opireeshiniin deesse(02) |  |
| 27 | Erga deessanii booda Gorsa/ barumsa mana yaalaa irraa fudhatanituu? | Eeyeen(01)  Miti (02) |  |
| 28 | Maddi odeeffannoo waa’ee Daa’ima hanga ji’a jahatti harma qofaa hoosisuu irraa argattan maali? | Ogeesa fayyaa (01)  Kutaa odeefanoo danbalii (Radiyoo, televezhinii)(02)  Garee gitaarra/Hiriyyaa irraa(03)  Garee shanee sirna/humna guddistu fayyaa(04) |  |
| 29 | Yoo maddi odeefannoo kee karaa danbalii ykn dubbisaa ta’ee isaa keessa karaa kamiin odeeffannoo argata? | Raadiyoo (01)  Televisizhinii (02)  Gaazexaa(03)  Interneetii (04) |  |
| **Gaaffilee Beekumsaa fi Ogummaa ilaalchisee** | | | |
| 30 | Erga deessee turtii yeroo hammami booda Daa’imakee harma hoosifte? | Yeroodhuma sanatti(01)  Saa’atii -----------booda(If <24 hr) (02)  Guyyaa------------booda(03)  Hin yaadadhu(04) |  |
| 31 | Daa’ima keessan guyyaa jalqabaa harma otoo hin hoosisiin dura nyaata/dhangala’aa kennitaniifii turtanii? | Eyyee (01)  Miti?lakki (02) | Yoo miti ta’e gara G-33darbi |
| 32 | Yoo kennittaniif ta’e maal keennitaniif? | Bishaan(01)  Dadhaa(02)  Aannan(03)  Kan biro-----------------------------(04) |  |
| 33 | Annan Harmaa kan jalqaba bahe elmitanii gattanii? | Eeyyee(01)  Miti/lakki(02) | Yoo miti ta’e gara G-35 darbi |
| 34 | Yoo Eelmitanii gattan ta’e maaliif? | ------------- |  |
| 35 | Ammayyu harma hoosisaa jirtuu? | Eeyyee(01)  Miti/lakki(02) | Eeyyee yoo ta’e gara G-37 ti darbi |
| 36 | Yoo miti ta’e maaliif? | -------- |  |
| 37 | Sa’aatii 24 darban kana keessatti yeroo meeqa hoosiftan? | -------------------- |  |
| 38 | Sa’aatii 24 darban kana keessatti harma irratti dabalataan nyaata /dhangal’aa kennitaniifii turtanii? | Eeyyee(01)  Miti/lakki(02) | Yoo miti ta’e gaaffii 36 fi 39 dhiisaa. |
| 39 | Yoo eeyyee ta’e maalfaa kennitaniif? | Aannan sa’aa(01)  Dhadhaa(02)  Bulbulamaa Sukkaaraa(03)  Daakuu aannanii Gabaa irraa bitamu(04)  Shoorbaa(axmiet) (05)  Kan biraa(ibsi)-----------------------(06) |  |
| 40 | Daa’ima keessaniif Umurii ji’a meeqaffaa isaa/ isheetti(maqaa) nyaata dabalataa kennuufi eegaltan? | -------------------- |  |
| 41 | Nyaata dabalataa kana daa’ima keessaniif keennuu maaliif barbaaddani | ---------------------------- |  |

| 42 | Daa’ima kee maalin afaanitti laattaf? | Fal’ana fayyadamuun(01)  Harkaan(02)  Xuuxxoon(03)  Warren armaan olii wal jijjiiruun(04) |  |
| --- | --- | --- | --- |
| 43 | Daa’ima keessaniif nyaata madaalamaa irra jireessatti maal irraa qopheessituuf? | Midhaan dheedhii irraa(01)  Midhaan kanneen akka baaqelaa, ataraafi kkf. Irraa(02)  Midhaan dheedhii fi kanneen akka baaqelaa, ataraa,kkf irraa(03) |  |
| 44 | Yeroo soomii daa’ima keef nyaata bineeld fi biqilaa irraa argamu waliin makte ni qopheessitaafi? | Eyyee (01)  Lakki(02) |  |
| 45 | Yoo qopheessineef sababni isaa maaliif? |  |  |
| 46 | Daa’ima keef nyaata nyaata burqaan isaa bineelda irraa fi biqilaa irraa waliin makte ni laattaaf | Eyyee(01)  Lakki (02) |  |
| 47 | Bifa kamiin nyaata daa’ima keef qopheessita. | Bifa dhangala’aa ,marqaa qal’aa tiin (01)  Nyaata furdaa fkn marqaa (02)  Nyaata jajjaboo(03) |  |
| 48 | Daa’ima keef nyaata warshaa irraa qophaa’ee dhufu ni kennitaafi (laattafi)? | Eyyee(01)  Lakki (02) | Eyyee yoo jette gaaffi itti aanu (46ffaa) deebisi. |
| 49 | Deebbiin kee eyyee yoo ta’e, nyaata madaalamaa qophaa’aa kam fayyadamta daa’ima kee sooruf? | Daakuu aannanii(01)  Aannan danfe (02)  midhaan dheedhii irratti hundaa’e(03)  fuduraa irratti kan hundaa’e (fkn. Mango, burtukana, kkf.) (04) |  |
