# Supplementary material for: Magnitude and determinants of appropriate complementary feeding practice among mothers of children age 6–23 months in Western Ethiopia
Source: PLoS One. 2020 Dec 31;15(12):e0244277. doi: 10.1371/journal.pone.0244277 (PMC7774947; doi:10.1371/journal.pone.0244277)
Supplement: Attachment — Submitted filename: Comment plose.pdf [file pone.0244277.s004.pdf]

Relevance to the scientific community

Overall, the study is well written and addresses its objectives

Abstract

Background

There are many studies conducted in Ethiopia in this area. Could you please justify your reason for conducting this study and what is really the contribution of this study for the existing literature?

Methods

In the **Sample size and sampling procedure section last sentence you said that ,,,** In the case of more than one eligible participant in the household, lottery method was used to select one..,, Who were the eligible participants in the house hold??? Have you interviewed other than the mother? What is the impact of using participants other than the mother???

Data collection procedures

Interview is associated with recall bias..How did you addressed this bias?????

Did you include exhaustive list of possible predictors..???

Result

Discussion

Discussion section The 4th paragraph the last two statements say.. ,,our finding shows that children in the study area as well as in Ethiopia in general are not getting adequate energy and nutrients compared to children in many African and Asian countries. you are trying to generalize to the country. **Is it possible to generalize your finding to the country????**

What are the limitations of your study?

Conclusion

They are drown from the result

Is the manuscript technically sound, and do the data support the conclusions?yes

Has the statistical analysis been performed appropriately and rigorously?yes

Have the authors made all data underlying the findings in their manuscript fully available? yes
Is the manuscript presented in an intelligible fashion and written in standard English?yes
