# Supplementary material for: Magnitude and determinants of appropriate complementary feeding practice among mothers of children age 6–23 months in Western Ethiopia
Source: PLoS One. 2020 Dec 31;15(12):e0244277. doi: 10.1371/journal.pone.0244277 (PMC7774947; doi:10.1371/journal.pone.0244277)
Supplement: Attachment — Submitted filename: Response to reviewers.doc [file pone.0244277.s005.doc]

**Response to reviewers**

Manuscript title: Magnitude and determinants of appropriate complementary feeding practice among mothers of children age 6-23 months in Western Ethiopia: community based cross sectional study

| **Editor/ Reviewer** | **Editor’s /Reviewers’ comments** | **Authors’ response** | **Remark** |
| --- | --- | --- | --- |
| **Editor** | Please ensure that your manuscript meets PLOS ONE's style requirements, including those for file naming | By now, we have followed the submission guideline and revised the manuscript accordingly. |  |
|  | Please include additional information regarding the survey or questionnaire used in the study and ensure that you have provided sufficient details that others could replicate the analyses. For instance, if you developed a questionnaire as part of this study and it is not under a copyright more restrictive than CC-BY, please include a copy, in both the original language and English, as Supporting Information. Moreover, please include more details on how the questionnaire was pre-tested, and whether it was validated. | The survey questionnaire in its English version and in the local language (Afan Oromo) is included as supporting information. Thanks |  |
|  | Please amend either the title on the online submission form (via Edit Submission) or the title in the manuscript so that they are identical. | This is addressed in the revised submission. |  |
|  | PLOS requires an ORCID iD for the corresponding author in Editorial Manager on papers submitted after December 6th, 2016. Please ensure that you have an ORCID iD and that it is validated in Editorial Manager. | ORCID iD is used in the revised submission. |  |
|  | Please amend your list of authors on the manuscript to ensure that each author is linked to an affiliation. Authors’ affiliations should reflect the institution where the work was done (if authors moved subsequently, you can also list the new affiliation stating “current affiliation:….” as necessary). | This comment is kindly addressed. |  |
|  | We note you have included a table to which you do not refer in the text of your manuscript. Please ensure that you refer to Table 5 in your text; if accepted, production will need this reference to link the reader to the Table. | Thanks. This was an error. It was mentioned as table 4 in the text but it was table 5 | Please see line 193 |
|  | Your ethics statement must appear in the Methods section of your manuscript. If your ethics statement is written in any section besides the Methods, please move it to the Methods section and delete it from any other section. Please also ensure that your ethics statement is included in your manuscript, as the ethics section of your online submission will not be published alongside your manuscript. | We have brought the ethics statement in the methods section in the revised manuscript. | Please see lines 141-146 |
| Reviewer-1 | The arguments in this study are very weak in informing policy. | Much information is included in the background of the study based on the comments given. Besides, rather than informing policy, the statement is changed as designing appropriate intervention for the study area. | Please see lines 80-81 |
|  | The grammar is generally poor and requires an English language editor’s services. | We tried to edit the manuscript as much as possible to address this comment. |  |
|  | There are WHO guidelines on breastfeeding for HIV exposed infants and the rest of the population that are clear when each feeding method can be initiated and discontinued, however, this study has not referred to these. | WHO’s recommendation about appropriate complementary feeding for breastfed and non-breastfed children is now included. | Please see lines 44-52 |
|  | If a researcher chose to read only the abstract, then they would consider the paper very inadequate in addressing the gap in research. | The gap is now included in the abstract. | Please see lines 10-12 |
|  | The magnitude of appropriate complementary feeding practice in the study area was very low”. What would you consider low. | Only 9.9% of mothers were practicing appropriate complementary feeding practice in the study area. We think that this number can be considered as very low.  However, to avoid ambiguity, we modified the statement as ‘Only one tenth of mothers practiced appropriate complementary feeding’. | Please see lines 27 |
|  | There are certain questions one would like to answer: What is complementary feeding practice? Is it a collection of different activities or it is one event? This is not clear in the background. | The definition of complementary feeding is now included in the manuscript including WHO’s indicators of appropriate complementary feeding practice. | Please see lines 38-52 |
|  | The authors will do well to give a clear explanation of the food types as they appear in the national guidelines and also based on food types in the region. Any brief explanation of these groups and maybe what do they comprise of in Ethiopia. It would help also to provide information on guidelines relating to initiation of complementary foods in Ethiopia and also speaking to WHO guidelines. | This comment is addressed by mentioning the national strategy which follows WHO’s recommendation to improve nutrition of young children in Ethiopia | Please see lines 54-59 |
|  | What is context specific information? | This was to mean that information specific for the study area is required to design appropriate intervention. But now, we removed the phrase as it may be ambiguous for readers. |  |
|  | Lines 50-51-the authors collected data to determine the magnitude of appropriate complementary feeding practice and its determinants in Western Ethiopia to guide policy actions in the area. This aim is two pronged because the authors can come up with just results on magnitude of complementary feeding practice in the population of interest….but also they can establish determinants of complementary feeding practice. Handling the study in this way made it difficult for the authors to make scientifically based conclusions as shown later in the comments. Either one of the issues studied separately would provide evidence to inform policy. For this study, the main outcome measure (lines 76 & 77) do not relate with the aim of the study. Therefore, the authors were not mindful of the importance of answering the research question adequately and might benefit from recasting the overall aim to tally with what the data is describing. | In our study, we determined the **magnitude** of appropriate complementary feeding practice and its **determinants.** The main outcome variable of the study was **appropriate complementary feeding practice.** The title of the manuscript is now modified accordingly and rather than informing policy, the study results can be used to design proper intervention in the study area and this is mentioned in the revised manuscript. |  |
|  | Here it would help to describe what constitutes appropriate complementary feeding and its components | The concept of appropriate complementary feeding practice is described now by refereeing WHO’s definition of appropriate complementary feeding practice using its four indicators. | Please see lines  44-52 |
|  | Under **variables** of the study, the authors mention that the mothers identified for this study should have ANC visits, delivered at the health facility and attended PNC with additional information on feeding counseling. The link is unclear, but how do you link this care to magnitude of appropriate complementary feeding? | Mothers were not expected to fulfill the criteria rather these were the variables which would have associations with the practice of complementary feeding practice (possible determinants). Their association with the outcome variable (appropriate complementary feeding practice) was thus examined. |  |
|  | **Data collection procedures:** Instead of saying diploma or BSc nurses, one would think describing their skills is better than their qualification. | The data collectors and supervisors had prior experience in data collection. This is mentioned in the revised manuscript. | Please see lines 117-120 |
|  | Result:  This section of the manuscript should be reorganized based on the overall aim after recasting it. | Since we have revised the title and aim of the study, we think that the way the result is presented in the manuscript looks okay. |  |
|  | **DISCUSSION**  This statement should open with the main outcome of the study to see that the research question has been answered. | This comment is addressed now. We started by stating the magnitude of appropriate complementary feeding practice in the study area | Please see lines 211-217 |
|  | Lines 208-209-This statement contradicts with the WHO recommendation of exclusive breastfeeding up to six months. This is worrying. | In the manuscript lines 208-209 state about the following :  ‘On the other hand, the proportion of children who achieved the minimum acceptable diet in our study was lower than  findings reported from Sir Lanka, Bangladesh, Nepal and Northern Ghana[13-15,20].’  There is no any concept which contradicts with this statement. There seems a mismact in the lines and comments indicated. This appears also in some comments given the discussion part. However, we tried to address all the comments as much as possible. |  |
|  | Lines 209-201-What would you consider to be premature for your study? This comparison might not suffice because of differences in characteristics. | Premature is to mean early and the word is now replaced by early in the manuscript. | Please see line 223 |
|  | Lines 228-231: sentence is too long and needs recasting to make the message clear in this context. | This is kindly noted. Most statements in the discussion are rewritten now to make them short. | Please see the discussion part of our manuscript. |
|  | *Our finding shows that children in the study area as well as in Ethiopia in general are not getting adequate energy and nutrients compared to children in many African and Asian countries:* This sentence requires a source; I doubt the authors have authority to this information. | This comment is well noted. The statement is now modified and the finding is now used to comment the practice in the study area only | Please see lines 251-252 |
|  | Lines 256-257: This type writing is all over the discussion and it has become monotonous. The authors can vary the expression. | The discussion part is now modified based on this and other comments mentioned above. |  |
|  | If the idea is to improve the practices among the uneducated women, redesigning and packaging the intervention to suite this population then ties in with what authors earlier indicated in line 49-50 *‘context specific information to design appropriate interventions’* | The aim of the study was to determine the magnitude of appropriate complementary feeding practice and its determinants in the study area. Based on that, we determined the magnitude and in fact the result was similar with most previous studies in Ethiopia. Besides this, we examined the determinants. Factors or determinants which were negatively associated with appropriate complementary feeding practice were lower age of the child, birth order (being the first child) and mother’s literacy level.  Based on this finding, we recommended interventions such as nutrition education to be given focusing on participants with young children, mothers who gave birth for the first time, and uneducated mothers in the study area.  In fact, some of the determinants such as mothers’ education were also reported in other Ethiopian studies. |  |
| **Reviewer-2** | There are many studies conducted in Ethiopia in this area. Could you please justify your reason for conducting this study and what is really the contribution of this study for the existing literature? | As mentioned, there are a number of studies on complementary feeding practice in Ethiopia. However, there is no any study in the study area. Since there are regional differences in culture and food practices in Ethiopia, we thought the research is worth investigating. |  |
|  | In the **Sample size and sampling procedure section last sentence you said that ,,,** In the case of more than one eligible participant in the household, lottery method was used to select one..,, Who were the eligible participants in the house hold??? Have you interviewed other than the mother? What is the impact of using participants other than the mother??? | The eligible participants were mothers with children 6-23 months old. In cases where there were two children in this age range (6-23months), one child was selected with lottery method. | Please see lines 101-102 |
|  | Interview is associated with recall bias.  How did you address this bias????? | This is totally correct.  To minimize recall bias, data collectors were well trained during the training period about probing the participants by explaining the questions which are susceptible to recall bias in many ways to help participants report accurate recalls. Participants were also given enough time to think and respond to the questions. |  |
|  | Discussion section The 4th paragraph the last two statements say.. ,,our finding shows that children in the study area as well as in Ethiopia in general are not getting adequate energy and nutrients compared to children in many African and Asian countries. You are trying to generalize to the country. **Is it possible to generalize your finding to the country????** | The statement is now revised and the finding is used to comment the practice in the study area only. | Please see lines 251-252 |
|  | What are the limitations of your study? | The limitations of the study are included in the study.  Our study had two major limitations. The cross sectional nature of the study prevents it to establish a cause and effect relationship. Though a number of measures were taken, there might be a possibility to recall bias some variables such as time to introduction of complementary foods and type of foods given to the child the day before data collection. | Please see lines 271-274 |
