# Supplementary material for: Magnitude and determinants of appropriate complementary feeding practice among mothers of children age 6–23 months in Western Ethiopia
Source: PLoS One. 2020 Dec 31;15(12):e0244277. doi: 10.1371/journal.pone.0244277 (PMC7774947; doi:10.1371/journal.pone.0244277)
Supplement: Attachment — Submitted filename: PLOS ONE-PONE-D-20-06067-Reviewer comments.docx [file pone.0244277.s003.docx]

PLOS ONE

Determinants of appropriate complementary feeding practice among mothers of children age 6-23 months in Western Ethiopia

--Manuscript Draft--

| Manuscript Number: | PONE-D-20-06067 |
| --- | --- |
| Article Type: | Research Article |
| Full Title: | Determinants of appropriate complementary feeding practice among mothers of children age 6-23 months in Western Ethiopia |
| Short Title: | Determinants of appropriate complementary feeding practice among mothers of children age 6-23 months |
| Corresponding Author: | Hirut Assaye Cherie, PhD  Bahir Dar University Institute of Technology Bahir Dar, ETHIOPIA |

**REVIEWER COMMENTS**

**GENERAL COMMENTS**

Determining the complementary feeding practices is critical for children aged six months and above. Therefore, this study is very critical in addressing behaviour change regarding best practices among the population of interest. This being the case, the arguments in this study are very weak in informing policy.

The grammar is generally poor and requires an English language editor’s services.

There are WHO guidelines on breastfeeding for HIV exposed infants and the rest of the population that are clear when each feeding method can be initiated and discontinued, however, this study has not referred to these.

**ABSTRACT**

If a researcher chose to read only the abstract, then they would consider the paper very inadequate in addressing the gap in research.

Lines 23-24, the authors said: “The magnitude of appropriate complementary feeding practice in the study area was very low”. What would you consider low.

**BACKGROUND**

Much as the background needs to be brief, it has very little to reflect the gap that this research is addressing.

There are certain questions one would like to answer: What is complementary feeding practice? Is it a collection of different activities or it is one event? This is not clear in the background.

The authors will do well to give a clear explanation of the food types as they appear in the national guidelines and also based on food types in the region. Any brief explanation of these groups and maybe what do they comprise of in Ethiopia. It would help also to provide information on guidelines relating to initiation of complementary foods in Ethiopia and also speaking to WHO guidelines.

What is context specific information?

**OVERAL AIM**

Lines 50-51-the authors collected data to ***determine*** the magnitude of appropriate complementary feeding practice and its ***determinants*** in Western Ethiopia to guide policy actions in the area.

This aim is two pronged because the authors can come up with just results on ***magnitude of complementary feeding practice*** in the population of interest….but also they can ***establish determinants of complementary feeding practice***.

Handling the study in this way made it difficult for the authors to make scientifically based conclusions as shown later in the comments. Either one of the issues studied separately would provide evidence to inform policy.

For this study, the main outcome measure (lines 76 & 77) do not relate with the aim of the study. Therefore, the authors were not mindful of the importance of answering the research question adequately and might benefit from recasting the overall aim to tally with what the data is describing.

**METHODOLOGY**

Here it would help to describe what constitutes appropriate complementary feeding and its components

Under **variables** of the study, the authors mention that the mothers identified for this study should have ANC visits, delivered at the health facility and attended PNC with additional information on feeding counselling. The link is unclear, but how do you link this care to magnitude of appropriate complementary feeding?

**Data collection procedures:** Instead of saying diploma or BSc nurses, one would think describing their skills is better than their qualification.

**RESULTS**

This section of the manuscript should be reorganized based on the overall aim after recasting it.

**DISCUSSION**

This statement should open with the main outcome of the study to see that the research question has been answered.

Lines 208-209-This statement contradicts with the WHO recommendation of exclusive breastfeeding up to six months. This is worrying.

Lines 209-201-What would you consider to be premature for your study?

This comparison might not suffice because of differences in characteristics.

Lines 228-231: sentence is too long and needs recasting to make the message clear in this context.

*Our finding shows that children in the study area as well as in Ethiopia in general are not getting adequate energy and nutrients compared to children in many African and Asian countries:* This sentence requires a source; I doubt the authors have authority to this information.

Lines 256-257: This type writing is all over the discussion and it has become monotonous. The authors can vary the expression.

**CONCLUSION**

Lines 271-273: When you compare this statement with the aim of the study and the background, the authors appear to be contradicting themselves or tying themselves. Their study does not appear to be different from the survey they referred to in the background.

The recommendation: ‘*Interventions on appropriate complementary feeding should therefore target uneducated mothers, mothers who gave birth for the first time and those with young children’.*

If the idea is to improve the practices among the uneducated women, redesigning and packaging the intervention to suite this population then ties in with what authors earlier indicated in line 49-50 *‘context specific information to design appropriate interventions’*
